# Audible Sound Stress Alters Behavior and Gene Transcription, and Negatively Impacts Development, Survival and Reproductive Fitness in *Spodoptera frugiperda*

**DOI:** 10.3390/insects17050467

**Published:** 2026-04-30

**Authors:** Chao-Yang Duan, Yun-Ju Xiang, Jun-Bo Li, Jun-Zhong Zhang, Da-Ying Fu, Wei Gao, Jin Xu

**Affiliations:** 1 Yunnan Provincial Key Laboratory for Conservation and Utilization of In-Forest Resource, Southwest Forestry University, Kunming 650224, China; duanchaoyang189@163.com (C.-Y.D.); xyj995999@163.com (Y.-J.X.); 13187858934@163.com (J.-B.L.); zhangjunzhong@foxmail.com (J.-Z.Z.); axl514@126.com (D.-Y.F.); 2 Key Laboratory for Forest Resources Conservation and Utilization in the Southwest Mountains of China, Ministry of Education, Southwest Forestry University, Kunming 650224, China; 3 Wenshan Plant Protection and Quarantine Station, Wenshan 663099, China

**Keywords:** *Spodoptera frugiperda*, sound stress, development, reproductive fitness, transcription

## Abstract

Moth auditory systems, evolutionarily adapted and structurally diverse with ultrasonic sensitivity, underpin the development of acoustic-based pest management strategies. Here, based on hypotheses derived from previous findings, we tested whether and how audible sounds (music, bird chirp, noise; 0.25–1 kHz, 80/120 dB) affect the development, survival, behavior and fecundity, as well as the molecular responses, using both short-term and long-term exposure (three successive generations) experimental designs. Behavioral assays showed dose-specific responses: high-intensity (120 dB) bird chirp and noise suppressed larval and adult activity, while low-intensity (80 dB) counterparts promoted larval crawling. Long-term exposure revealed that bird chirp and noise significantly impaired fitness, reducing larval/pupal body weight, pupation/eclosion rates, and egg hatching rate, with 120 dB noise exerting the strongest effects; 80 dB music showed neutral or positive impacts. Transcriptomic analysis identified 71–235 differentially expressed genes (DEGs) across treatment groups, with bird chirp and noise inducing more downregulated DEGs related to metabolism, immunity, and development. Notably, all cuticle-related DEGs in the 80 dB noise group and 53.2% in the 120 dB noise group were upregulated, suggesting stress-induced cuticular remodeling. GO/KEGG enrichment indicated distinct patterns: 80 dB music, bird chirp and 120 dB noise groups only had downregulated DEGs enriched in certain terms/pathways, mainly associated with cellular components; the 80 dB noise group had upregulated DEGs enriched in sensory, cuticle, metabolism and longevity-related terms/pathways, and downregulated DEGs in metabolism and human disease-related terms/pathways. Analysis of the expression patterns of all the longevity pathway-related genes suggested that sound stress induces lifespan regulation in this insect. These findings clarify *S. frugiperda’s* multidimensional responses to audible sound, providing a foundation for sound-based pest management.

## Simple Summary

This study investigated how audible sounds (0.25–1 kHz, 80/120 dB: music, bird chirp, noise) affect *Spodoptera frugiperda* via short/long-term (three generations) exposure. Behavioral responses were dose-specific: 120 dB bird chirp/noise inhibited larval/adult activity, while 80 dB versions boosted larval crawling. Long-term exposure to bird chirp/noise severely impaired the pest’s fitness, reducing body weight, pupation/eclosion and egg hatching rates (120 dB noise had the strongest effect), whereas 80 dB music was neutral or beneficial. Transcriptomic analysis detected 71–235 differentially expressed genes per group; bird chirp/noise downregulated more metabolism, immunity and development-related genes, and noise induced cuticular remodeling via upregulated cuticle-related genes. Gene function enrichment analysis showed that sound stress-induced upregulated genes were enriched in sensory, cuticle, metabolism and longevity-related terms/pathways, while downregulated genes were enriched in cellular components, metabolism and human disease-related terms/pathways. These findings elucidate *S. frugiperda*’s responses to audible sound stress, laying a basis for acoustic-based pest management.

## 1. Introduction

*Spodoptera frugiperda* (Lepidoptera: Noctuidae), the fall armyworm, is a highly destructive, long-distance migratory agricultural pest native to the Americas [1]. Since its first detection in southwestern China in late 2018, the pest has rapidly spread across extensive regions of the country thereafter [2,3]. It causes massive economic losses to maize production, with the potential annual economic loss in China estimated at $17.3–52.1 billion [4]. In addition, its severe foliar damage, high fecundity, and strong pesticide resistance have induced the overuse of chemical pesticides, even prohibited and hazardous ones [5,6,7,8], which calls for environmentally friendly management strategies for effective control [1,8].

Insect auditory systems are remarkable examples of evolutionary adaptation, shaped by ecological pressures like predation avoidance and intraspecific communication [9]. Unlike vertebrate ears, insect auditory organs vary widely in different taxa, such as tympana in many orders (e.g., Lepidoptera, Orthoptera and Mantodea), antennal organs in honeybees, tibial organs in cockroaches, and abdominal organs in Pneumoridae [9,10]. Moth ears, namely tympanal organs, exhibit remarkable diversity in their anatomical placement and morphological features, yet share highly similar threshold curves; consistently, they are most sensitive to ultrasonic frequencies, with the best frequencies ranging from approximately ~20 to 60 kHz for the majority of species, which has evolved to achieve optimal sensitivity within the bat echolocation frequency range [10,11]. Lepidopteran larvae and pupae do not possess dedicated tympanal auditory organs, yet their immature life stages are able to perceive environmental acoustic signals through non-specialized sensilla [12,13]. Given the critical role of auditory function in insect survival and reproduction, acoustic-based methods have been widely explored for the trapping or detection of insect pests, as well as for altering their behavior by interfering with interspecific communication [14,15].

Previous studies also demonstrated that predators’ sounds and anthropogenic noise can negatively affect the behavior and physiology of insects, suggesting their potential utility in developing environmentally friendly pest control strategies [11,15,16,17]. For example, in the beet armyworm *Spodoptera exigua*, bat echolocation calls reduced the activity and reproductive output and altered larval metabolism, illustrating the non-consumptive potential of ultrasound for eco-friendly pest control [17]. Anthropogenic noise from sources such as construction and traffic generates low-frequency, high-amplitude sounds that can propagate over long distances through the air and various substrates, thereby overlapping with and disrupting critical biotic acoustic cues that govern key ecological processes [11,18]. For instance, traffic systems generate noise with frequency ranges spanning 10–10,000 Hz, with particularly high amplitudes at the frequency ranges that arthropods depend on (typically < 1000 Hz) [19]. As a result, anthropogenic noise can impair arthropods’ ability to detect and process the acoustic cues and signals that mediate behavioral decision-making during interspecific and intraspecific interactions [17,19,20,21,22]. A previous study in the green peach aphid, *Myzus persicae*, found that acoustic stimuli (five frequencies within 100–10,000 Hz, and three intensity levels within 60–90 dB) significantly reduced its feeding and honey dew production [23]. In ants, rhythmic noise (42 and 200 beats per minute) disrupted their orientation and social interaction, whereas the smooth sound of flowing water had either neutral or beneficial effects [24]. Recently, a study on crickets showed that traffic noise and white noise impair females’ ability to prefer higher-quality male mating songs, resulting in suboptimal mating decisions that could reduce fitness and weaken sexual selection [25]. Agah-Manesh et al. [16] tested ultrasound as physical control for *Sesamia cretica* (pink stalk borer) and identified 37.5–39.5 kHz as the most repellent frequencies, which reduced immature longevity, survival, and fecundity. Therefore, it is widely accepted that acoustic stress can adversely impact the survival and reproduction of insects. However, evidence and our understanding of this issue, particularly its underlying regulatory mechanisms, remain limited relative to the vast diversity of insect taxa and their highly diverse auditory systems.

When insects face environmental stresses such as heat and chemicals, they activate a coordinated survival response, such as adjusting behavior and metabolism, producing protective compounds like heat-shock proteins to prevent cellular damage. Crucially, these reactions are driven by rapid genetic changes; specific genes, particularly those involved in detoxification, thermal tolerance, and immune defense, are switched on. This swift molecular and systemic adaptation allows insects to withstand short-term threats [26,27,28] and can even lead to long-term evolutionary adaptations in populations [29,30,31]. These responses reduce acute toxic damage, whereas prolonged and persistent stressor exposure typically impairs fitness, owing to the disruption of cellular homeostasis, oxidative damage, protein misfolding, and organelle turnover, as well as impairments to the functions of proteins and other molecules [26,27,31,32,33,34]. The morphological and physiological consequences of acoustic overstimulation in insects have been studied extensively, but little is known about the molecular consequences of acoustic trauma. In locust *Schistocerca gregaria*, noise exposure impacts resulted in hearing loss, probably due to the decrease in the ability to transduce sound of the auditory receptors [35]. A later study in *S. gregaria* further demonstrated that noise exposure induced the upregulation of genes responsible for cuticular construction in Müller’s organs, whereas downregulation in many genes was involved in lipid and protein storage and metabolism [36].

In *S. frugiperda*, a previous study constructed a brain transcriptome of male moths exposed to bat ultrasound, and identified 290 differentially expressed transcripts that related to neurotransmitter metabolism, mitochondrial metabolism, antioxidant enzyme activity, heat shock protein activity, actin cytoskeleton dynamics, chromatin binding, methylation, cilia development, and signaling pathways [37]. As mentioned above, lepidopteran larvae lack specialized tympanal auditory organs but detect environmental acoustic signals via non-specialized sensilla. However, possible responsive gene expression induced by sound stress has not been tested in the larval stage of Lepidoptera and other insect taxa.

Moreover, quite a number of studies have investigated the responses of *S. frugiperda* to biotic and abiotic stresses across behavioral, physiological, and molecular levels related to stress response and resistance, development, reproduction and survival, providing a solid foundation for exploring the effects of acoustic stress on the physiological and molecular mechanisms of this pest species [27,28,31,38]. Based on above findings in moths and other insects, we hypothesize that: (a) both larvae and adults can detect audible sound stimuli; (b) persistent audible sound stimuli can negatively affect the survival and reproduction; (c) daytime sound exposure and nighttime sound exposure may result in different effects; (d) multigenerational sound exposure may induce acoustic acclimation; and (e) sound stress alters the expression of genes associated with cuticular structure, stress response and tolerance, development, and survival in *S. frugiperda* larvae. To systematically test these hypotheses, we designed a series of controlled laboratory experiments with standardized acoustic treatments: three sound types (rhythmic music, sparrow chirp, white noise; 0.25–1 kHz), two sound pressure levels (80 dB and 120 dB), and dual photoperiod exposure regimes (daytime vs. nighttime application). We comprehensively evaluated the effects of short-term sound exposure on immediate behavior of larvae and adults, as well as long-term chronic exposure on development and reproduction across three successive full life-cycle generations. Moreover, naïve fourth-instar larvae were subjected to short-term sound stresses, and then RNA-seq was performed to characterize their differential gene expression patterns, aiming to reveal the phenotypic plasticity and potential molecular mechanisms underlying insect responses to acoustic stress.

## 2. Materials and Methods

### 2.1. Insects

*S. frugiperda* larvae were collected from corn plants in a corn field near Dongchuan Town, Yunnan Province of China. Collected larvae were reared on an artificial diet [39] under controlled environmental conditions: temperature of 27 ± 1 °C, relative humidity of 60–80%, and a 14:10 h light/dark photoperiod. Mature pupae were sexed according to abdominal morphological characteristics [40] and then segregated into male and female groups for separate caging. Adult eclosion was monitored daily, and newly eclosed moths were individually caged by sex to ensure uniform age and virginity. All adult moths were provided with a 10% honey solution for nutrition.

### 2.2. Effect of Sound Exposure on the Behavior of Larvae and Adults

The sounds and exposure protocols were determined based on previous studies [16,23,25] and our preliminary experiments. Three sounds, including one music (piano music, One Summer’s Day, Joe Hisaishi), one bird chirp (recorded sparrow chirps), and one noise (recorded sound of a ceramic basin rubbing against the concrete floor) were used for the following treatments.

The 80 dB and 120 dB of the above music, bird chirp, and noise were used for behavioral tests, and no sound playing (silence) was used as controls. The sounds were played by using an Aige Q93 speaker (Shenzhen Jiayu Audio Electronics Co., Ltd., Shenzhen, China) and the volume (dB) and frequency (Hz) were measured using an Aihua AWA6228 multifunctional sound meter (Hangzhou Aihua Instrument Co., Ltd., Hangzhou, China). All behavioral tests were performed during the scotophase under the controlled environmental conditions described above. A 15 W red light served as the illumination source for observations.

To test the effect of sound on the behavior of larvae, the fourth-instar larvae (larvae at this stage are behaviorally active, and sufficiently large for easy manipulation) were randomly selected from the colony and divided into groups for the following tests. Before testing, a larva was caged in a plastic box that was covered with nylon mesh (25 cm long, 15 cm wide, 8 cm high) and was placed 0.5 m directly in front of the speaker and maintained for 0.5 h before playing the sound. After playing the sound, the number of crawling events and crawling distance of the larva within 0.5 h were recorded. One crawling event was determined from the start to a stop of the crawl and remains motionless within 10 s after stopping. One larva was used as a replicate and each sound treatment used 12 replicates. Sound pressure level (SPL) and frequency variability of each sound treatment across the box were measured at six positions within the box and the results are presented in Table S1. Briefly, the 80 dB music treatment exhibited an SPL range of 76.8–84.2 dB and a frequency range of 0.25–1 kHz within the box, 120 dB music had an SPL of 116.8–122.2 dB and a frequency of 0.25–1 kHz; 80 dB bird chirp had an SPL of 76.3–84.5 dB and a frequency of 4–8 kHz, 120 dB bird chirp had an SPL of 116.5–122.5 dB and a frequency of 4–8 kHz; 80 dB noise had an SPL of 77.3–84.1 dB and a frequency of 4–4.5 kHz, 120 dB noise had an SPL of 114.4–127.8 dB and a frequency of 4–4.5 kHz.

Our preliminary study showed that caged adult moths usually remain stationary regardless of being exposed to acoustic environments or kept in quiet conditions; therefore, we adopted an alternative approach to verify the effects of sound on adult behaviors. The 1 d old female adults were randomly selected from the colony and divided into groups for the following tests. Before testing, a cage covered with nylon mesh (45 cm long, 35 cm wide, 35 cm high) was placed 0.5 m directly in front of the speaker. After playing the sound, a caged adult was introduced into the test cage and the activity duration (from active, including walking, flying or funning the wings, to stationary) was recorded as the initial activity duration. After being stationary for more than 10 s, the adult was stimulated to become active again by gently knocking the cage, and the active duration was recorded as active duration of adult after mechanical stimulus. Repeat the mechanical stimulus tests five times for each adult. One adult was used as a replicate and each sound treatment used 12 replicates. SPL and frequency variability of each sound treatment across the cage were also measured at nine positions within the cage and the results are presented in Table S1. Briefly, the 80 dB music treatment exhibited an SPL range of 76.9–86.2 dB and a frequency range of 0.25–1 kHz within the cage, 120 dB music had an SPL of 116.8–121.1 dB and a frequency of 0.25–1 kHz; 80 dB bird chirp had an SPL of 77.1–84.1 dB and a frequency of 4–8 kHz, 120 dB bird chirp had an SPL of 116.9–123.8 dB and a frequency of 4–8 kHz; 80 dB noise had an SPL of 77.3–83.5 dB and a frequency of 4–4.5 kHz, 120 dB noise had an SPL of 111.4–121.0 dB and a frequency of 4–4.5 kHz.

### 2.3. Effect of Long-Term Sound Exposure on the Development and Reproduction of S. frugiperda

For the long-term test, sound treatments were applied to *S. frugiperda* across its entire life cycle (egg, larval, pupal, and adult stages) for three successive generations (G1–G3). Specifically, 80 dB of the music, 80 dB of the bird chirp, 80 dB of the noise, or 120 dB of the noise were played continuously for 10 h once each day either during the dark cycle (21:00 to 7:00) or the light cycle (7:00 to 21:00). To avoid interference, each sound treatment was carried out in a separate room under the controlled environmental conditions described above, with treatments being randomly assigned to different rooms. The sounds were played using a speaker as the above, which was placed 0.5 m directly in front of the insect containers. Sound exposure during the egg, larval and pupal stages was conducted in plastic boxes, while that for adults was applied in cages as described above, with identical acoustic parameters (Table S1). Normally reared insects (without sound exposure) were used as controls (CK).

At the beginning, 30 female and 30 male adults were randomly selected from the wild-type colony (no sound exposure) and paired. Eggs laid during the first three days were collected and assigned to five groups for the aforementioned sound treatments (first generation). From each treatment, 500 larvae were randomly chosen and reared in the box under continuous sound exposure until adult emergence. Subsequently, 30 females and 30 males were randomly selected from each treatment, paired in cages, and their eggs collected over the first three days. These eggs were subjected to identical sound treatments to establish the second generation, with 500 larvae per group reared to adulthood under the same sound conditions. This procedure was repeated for the third generation.

During each generation, the developmental durations of larvae and male and female pupae were recorded with a sample size of 60 insects (*n* = 60). The pupation rate of larvae and the eclosion rate of pupae were determined using five replicates per treatment (*n* = 5), with 24 larvae or 24 male or female pupae per replicate, respectively. The body weight of the sixth-instar larvae and 4 d old male and female pupae was measured using a Sartorius MSX electronic balance (accuracy: 0.0001 g; Sartorius, Göttingen, Germany), with a sample size of 60 insects (*n* = 60) per group.

To assess reproductive performance, 3 d old adults (reproductively mature) were paired (one pair per box) in plastic boxes (25 × 15 × 8 cm) and maintained until death. Each box was furnished with a zigzag-folded paper strip (15 × 20 cm) as an oviposition substrate and a 10% honey solution as a food source. All laid eggs were collected and incubated in Petri dishes (8.5 × 1.5 cm) under the aforementioned conditions. Hatching was recorded four days later. For each treatment, 20 pairs (*n* = 20) were used to quantify the total number of eggs laid and offspring larvae, and the egg hatching percentage.

### 2.4. Effect of Short-Term Sound Exposure on the Transcription of Larvae

#### 2.4.1. Treatment and Sample Collection

The fourth-instar larvae were randomly selected from the normally reared colony (without sounds exposure) and divided into groups for short-term (10 h) sound exposure during the dark cycle. The exposure treatments were: 80 dB music, 80 dB bird chirp, 80 dB noise, and 120 dB noise as the above. A control group (CK) received no sound exposure. For each treatment, three replicates (*n* = 3) of 15 insects each replicate were established. After exposure, larvae were immediately sampled and frozen in liquid nitrogen and stored at −80 °C.

#### 2.4.2. Sequencing and Mapping

Total RNA extraction was performed using TRIzol reagent (Invitrogen, Carlsbad, CA, USA). Concentration and purity were assessed using a spectrophotometer (Implen, Westlake Village, CA, USA) and a Qubit RNA Assay Kit (Life Technologies, Carlsbad, CA, USA), while integrity was verified with an RNA Nano 6000 Assay Kit (Agilent, Santa Clara, CA, USA). Sequencing libraries were constructed using the NEBNext Ultra RNA Library Prep Kit for Illumina (New England BioLabs, Ipswich, MA, USA) and sequenced on an Illumina HiSeq 4000 platform (Illumina, San Diego, CA, USA) to generate 125/150 bp paired-end reads.

For data processing, raw reads were first trimmed and filtered using fastp software (v0.23.4) to produce clean reads by removing adapters, undeterminable bases, and low-quality sequences. The Q20, Q30, and GC content of the clean data were then calculated. Subsequently, clean reads were mapped to the *S. frugiperda* reference genome (assembly AGI-APGP CSIRO Sfru_2.0) [41] using Hisat2 (v2.2.1) [42].

#### 2.4.3. Differential Expression and Functional Enrichment Analyses

Gene expression levels were quantified using transcripts per million (TPM). Differential expression analysis between treatments was performed with DESeq2 (v1.56.1) [43]. Differentially expressed genes (DEGs) were identified using a threshold of *q*-value < 0.05 (adjusted *p*-value) and |log2(fold change)| > 1 [44].

Functional enrichment analysis of the DEGs was conducted using two complementary approaches: Gene Ontology (GO) enrichment was assessed with the GOSeq program (v2.12) [45], and Kyoto Encyclopedia of Genes and Genomes (KEGG) pathway enrichment was analyzed using KOBAS software (v2.1.1) [46]. For both analyses, terms or pathways with a *q*-value < 0.05 and each term/pathway enriched by more than 3 DEGs were considered significantly enriched, and gene length bias was corrected in GOSeq.

#### 2.4.4. RNA-Seq Validation

RNA-seq validation was conducted by quantitative real-time PCR (qPCR). Following total RNA extraction (as described above), cDNA was synthesized with the PrimeScript RT reagent Kit (Takara, Beijing, China). Using the *RPL27* (LOC118269161) as the reference gene, qPCR amplification was performed in 25 μL reactions on a QuantStudio 7 Flex System (Thermo Fisher Scientific, Waltham, MA, USA) with gene-specific primers (Table S2). The thermal profile comprised an initial 95 °C for 30 s, followed by 40 cycles of 95 °C for 5 s and 60 °C for 30 s. Three biological replicates and three technical replicates per biological replicate were used for each sample. PCR efficiency was determined via standard curves generated from 5× serially diluted cDNA; amplification specificity was verified by melting curve analysis. Gene expression levels were calculated relative to the control using the 2^−ΔΔCT^ method [47].

### 2.5. Statistical Analysis

Data on developmental periods, body weight, reproduction, survival, and qPCR were analyzed using a one-way analysis of variance (ANOVA). Prior to ANOVA, the assumptions of homogeneity of variances and normality were assessed using Levene’s test and the Shapiro–Wilk test, respectively. Percentage data were subjected to arcsine square root transformation to meet these assumptions. Where ANOVA indicated significant effects, Fisher’s LSD test was implemented for post hoc multiple comparisons. The effect size *η*^2^ and its 95% CI of ANOVA were also reported. All analyses were performed in SPSS 16.0 (IBM Corp., Armonk, NY, USA). Statistical significance was defined at *α* < 0.05, and data are presented as mean ± standard error (SE).

## 3. Results

### 3.1. Effect of Sound Exposure on the Behavior of Larvae and Adults

Results showed that sound exposure significantly (ANOVA: *p* < 0.001; see Table S3 for detailed statistical results) affected the activities of larvae and adults.

**Figure 1:**
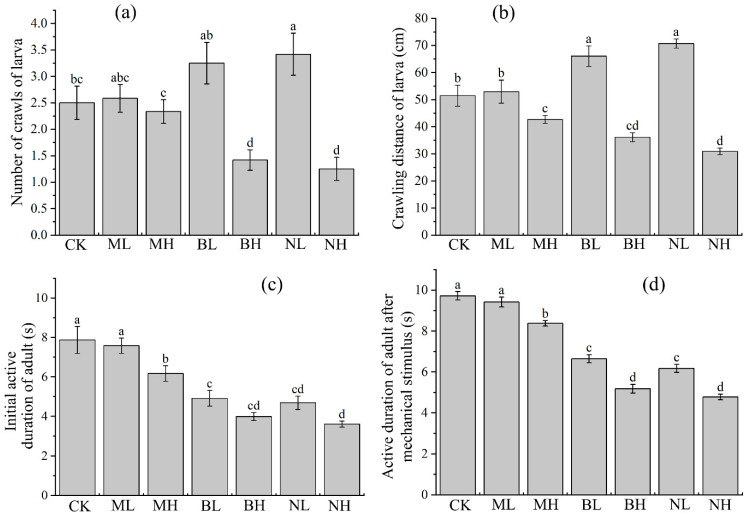
Effect of sound exposure on the activities of *S. frugiperda* larvae and adults. (**a**) Larval crawling events; (**b**) larval crawling distance; (**c**) adult initial activity duration; (**d**) adult active duration after mechanical stimulus. In each subgraph, different letters upon columns represent significant differences (*p* < 0.05) among treatments. CK refers to the control group, namely silence; ML refers to 80 dB music; MH refers to 120 dB music; BL refers to 80 dB bird chirp; BH refers to 120 dB bird chirp; NL refers to 80 dB noise; NH refers to 120 dB noise; the same as below.

For larvae, post hoc LSD test showed that, in comparison with controls: 120 dB bird chirp and noise significantly reduced the number of crawling events (*p* < 0.05), whereas 80 dB noise significantly increased the number of crawling events (*p* < 0.05), and 80 dB bird chirp, 80 and 120 dB music did not show significant effect (*p* > 0.05) (Figure 1a); 120 dB music, bird chirp and noise significantly reduced the crawling distance (*p* < 0.05), whereas 80 dB bird chirp and noise significantly increased the crawling distance (*p* < 0.05), and 80 dB music did not show significant effect (*p* > 0.05) (Figure 1b).

For adults, post hoc LSD test showed that, in comparison with controls: 120 dB music, 80 and 120 dB bird chirp and noise significantly reduced the initial active duration of adult (*p* < 0.05) and the active duration of adult after stimulus (*p* < 0.05), whereas 80 dB music did not show significant effect (*p* > 0.05) (Figure 1c,d).

### 3.2. Effect of Long-Term Sound Exposure on the Development and Reproduction of S. frugiperda

Sound exposures, either during dark cycle or light cycle, all significantly (ANOVA: *p* < 0.05; Table S3) affected the developmental periods, body size, survival and reproductive success of *S. frugiperda*, and showed a similar effect pattern in the three successive generations.

**Figure 2:**
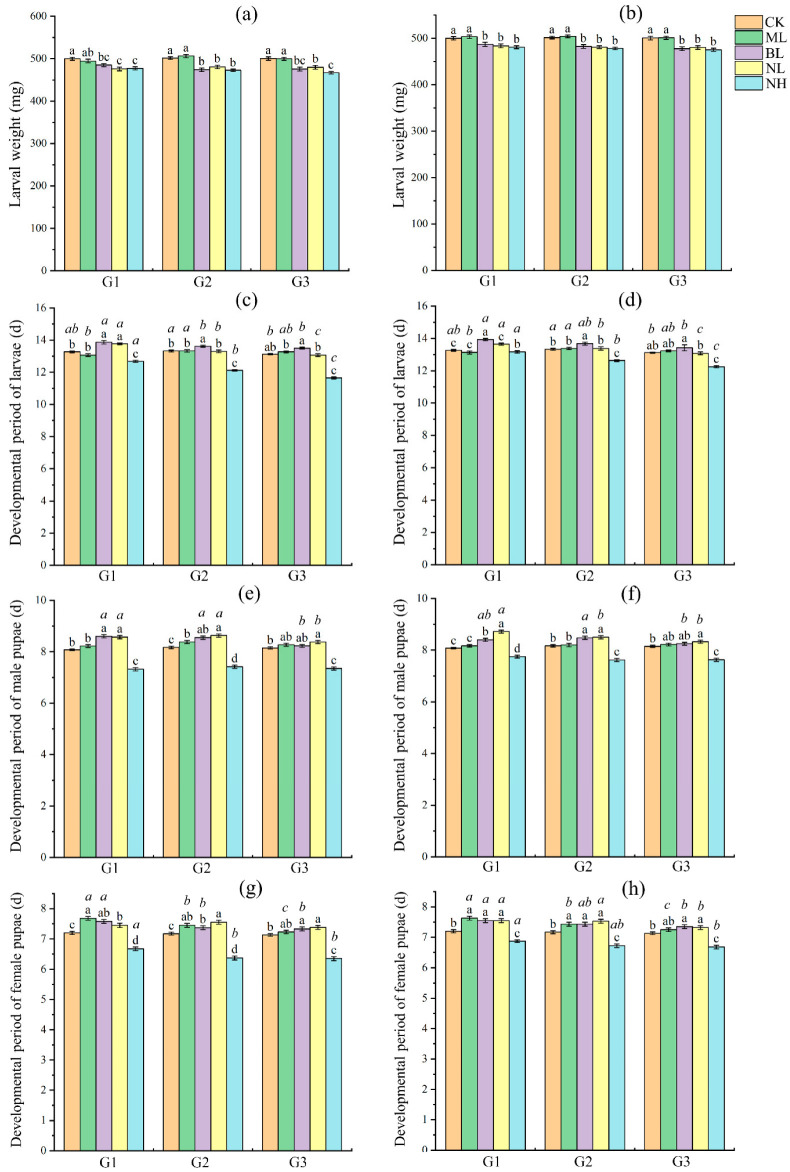
Effect of sound exposure on larval weight (**a,b**), developmental period of larvae (**c**,**d**), male pupae (**e**,**f**) and female pupae (**g**,**h**) of *S. frugiperda*. (**a**,**c**,**e**,**g**) represent sound exposure performed during the light cycle, whereas (**b**,**d**,**f**,**h**) represent sound exposure performed during the dark cycle. G1, G2 and G3 refer to the first, second and third generations, respectively. In each subgraph, different black regular letters upon columns represent significant differences among treatments of the same generation (*p* < 0.05); different italic letters above the columns of the same color indicate significant differences among generations under the same treatment (*p* < 0.05), while the absence of italic letter labels indicates no significant differences among generations under this treatment (*p* > 0.05). The same as below.

**Figure 3:**
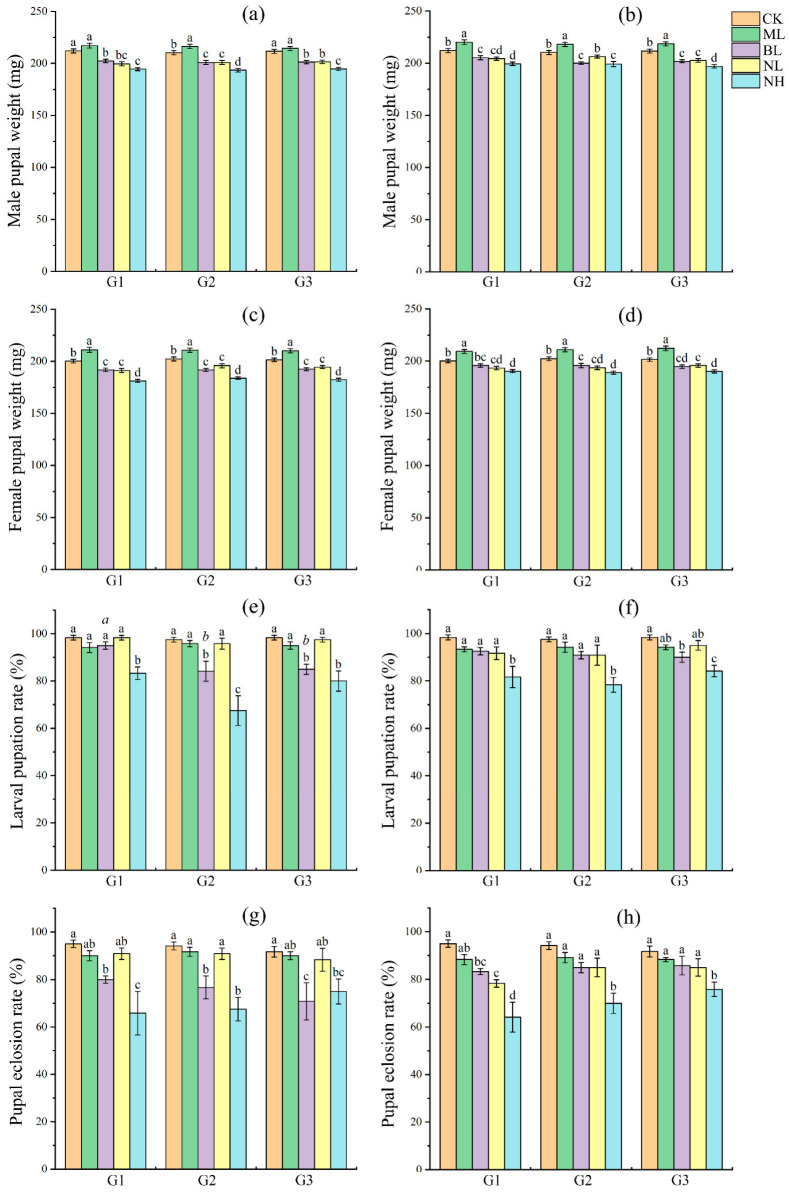
Effect of sound exposure on male pupal weight (**a**,**b**), female pupal weight (**c**,**d**) larval pupation rate (**e**,**f**), and pupal eclosion rate (**g**,**h**) of *S. frugiperda*. (**a**,**c**,**e**,**g**) represent sound exposure performed during the light cycle, whereas (**b**,**d**,**f**,**h**) represent sound exposure performed during the dark cycle. In each subgraph, different black regular letters upon columns represent significant differences among treatments of the same generation (*p* < 0.05); different italic letters above the columns of the same color indicate significant differences among generations under the same treatment (*p* < 0.05), while the absence of italic letter labels indicates no significant differences among generations under this treatment (*p* > 0.05).

For the development and survival, post hoc LSD test showed that, in comparison with controls: (1) 80 and 120 dB bird chirp and noises significantly decreased the body weight of larvae and male and female pupae in most cases (*p* < 0.05), while 80 dB music did not show a significant effect on larvae (*p* > 0.05) but a positive effect on pupae (*p* < 0.05) (Figure 2a,b and Figure 3a–d); (2) 120 dB noise significantly reduced the developmental period of larvae and male and female pupae in all tests (*p* < 0.05), whereas 80 dB noise and 80 dB bird chirp significantly prolonged the period in most cases (*p* < 0.05), and 80 dB music in most cases did not show significant effect (*p* > 0.05) and sometimes positive effects (*p* < 0.05) (Figure 2c–h); and (3) 80 dB bird chirp in many cases and 120 dB noise in all cases significantly reduced the larval pupation rate and pupal eclosion rate (*p* < 0.05), whereas 80 dB music in all cases and 80 dB noise in most cases did not show significant effect (*p* > 0.05) (Figure 3e–h). Significant differences among the three generations under the same treatment were also detected for several parameters: (1) In BL, NL, and NH, larval developmental period increased significantly with increasing generations (*p* < 0.05), whereas in CK and ML, it increased in G2 and then decreased in G3 (*p* < 0.05) (Figure 2c,d); (2) in most cases in NL and NH, the developmental period of male and female pupae increased significantly with increasing generations (*p* < 0.05) (Figure 2e–h); (3) in BL, larval pupation rate decreased significantly with increasing generations (*p* < 0.05) (Figure 3e).

**Figure 4:**
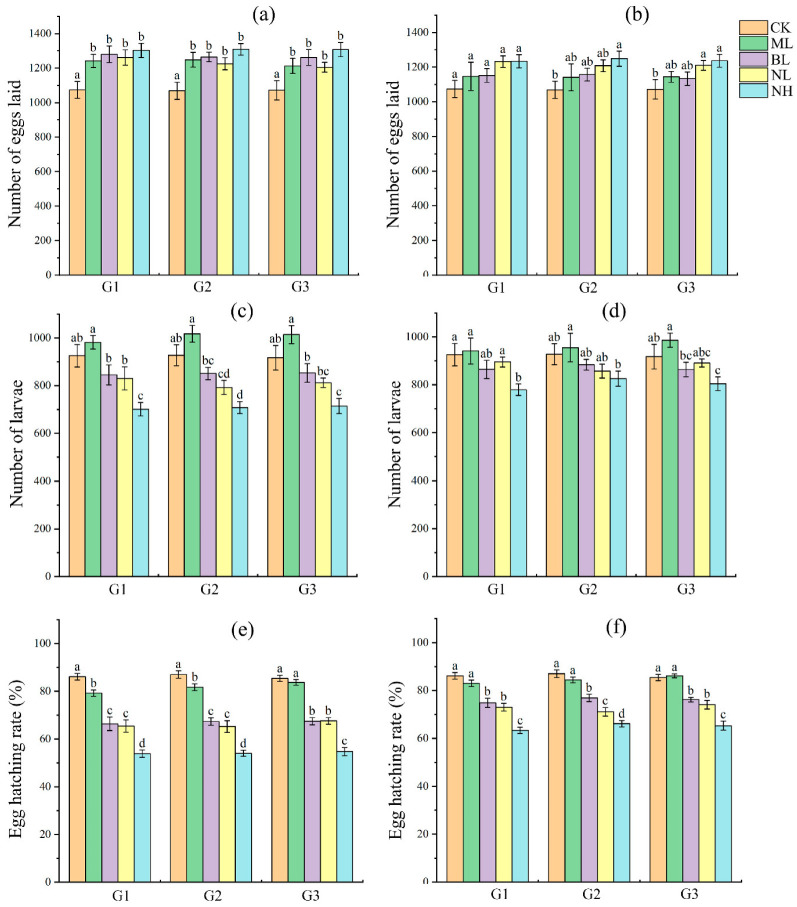
Effect of sound exposure on the reproductive fitness of *S. frugiperda*. (**a**,**c**,**e**) represent the number of eggs laid, number of larvae, and egg hatching rate, respectively, with sound exposure performed during the light cycle; (**b**,**d**,**f**) represent the number of eggs laid, number of larvae, and egg hatching rate, respectively, with sound exposure performed during the dark cycle. In each subgraph, different letters upon columns represent significant differences (*p* < 0.05) among treatments.

On the reproduction, post hoc LSD test showed that, in comparison with controls, (1) sound exposure all resulted in higher number of eggs laid, with most of them being significant (*p* < 0.05) (Figure 4a,b); (2) however, bird chirp and noises all resulted in lower number of offspring larvae, with most of them being significant (*p* < 0.05), and music always showed a relatively higher number of offspring larvae but not statistically significant in all cases (*p* > 0.05) (Figure 4c,d); (3) bird chirp and noise all resulted in significantly lower egg hatching rate (*p* < 0.05), while music did not show significant effect in most cases (*p* > 0.05) (Figure 4e,f). No significant differences in reproductive parameters were detected among the three generations under the same treatment.

### 3.3. Effect of Short-Term Sound Exposure on the Transcription of Larvae

#### 3.3.1. RNA Sequencing and Mapping

RNA-seq analysis of the 15 sequencing libraries yielded 40.3–52.6 million clean reads per library. The quality metrics of the sequencing data showed that the Q20, Q30, and genome-mapped ratios ranged from 98.65% to 98.74%, 95.73% to 96.00%, and 74.83% to 78.90%, respectively (Table S4). Pearson’s correlation coefficient analysis revealed weak correlations across different treatment groups but strong correlations among biological replicates (Table S5). In parallel, principal component analysis (PCA) demonstrated distinct clustering of biological replicate samples (Figure S1). Collectively, these results validated the high reproducibility of both the RNA-seq data and biological replicates in this study.

#### 3.3.2. Summary of Transcriptional Changes and DEG Annotation

A total of 71 (11 up, 60 down), 220 (9 up, 211 down), 199 (100 up, 99 down), and 235 (64 up, 171 down) DEGs (*q* < 0.05 and |log2FC| > 1) were identified in the ML vs. CK, BL vs. CK, NL vs. CK, and NH vs. CK groups, respectively (Figure S2). There were 51 common DEGs between ML vs. CK and BL vs. CK; 6 common DEGs between ML vs. CK and NL vs. CK; 51 common DEGs between ML vs. CK and NH vs. CK; 1 common DEG between BL vs. CK and NL vs. CK; 31 common DEGs between NL vs. CK and NH vs. CK; and 147 DEGs were shared between NH vs. CK and BL vs. CK (Figure S3). There were 48 common DEGs among ML vs. CK, BL vs. CK and NH vs. CK. No common DEGs were shared by all the four groups.

**Figure 5:**
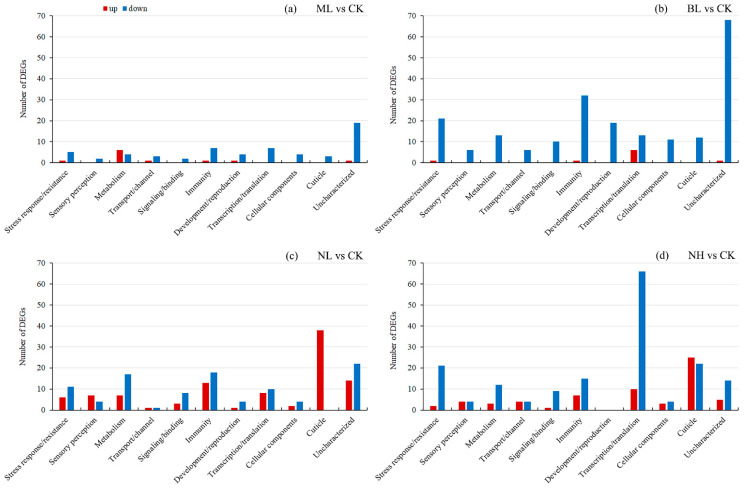
DEG annotation and classification. (**a**–**d**) represent DEGs of ML vs. CK, BL vs. CK, NL vs. CK, and NH vs. CK groups, respectively. Red columns denote the significantly upregulated DEGs, while blue columns denote the significantly downregulated DEGs.

The majority (581/725 = 80.1%) of DEGs (*q* < 0.05 and |log2FC| > 1) were successfully annotated (Table S6). To facilitate comprehensive understanding and summarization, all annotated DEGs were functionally clustered into 10 distinct collections (Figure 5). It is showed that the annotated DEGs from ML vs. CK group (51 annotated DEGs) and the BL vs. CK group (151 annotated DEGs) both had a dominant downregulation pattern across most functional categories (especially DEGs related to immunity and stress response/resistance), with only a small number of upregulated genes. In contrast, the NL vs. CK group (163 annotated DEGs) and NH vs. CK group (216 annotated DEGs) had a higher proportion of upregulated genes, with all cuticle-related DEGs in the NL vs. CK group being upregulated, while the NH vs. CK group had the largest number of DEGs related to transcription/translation and cuticle.

#### 3.3.3. Functional Enrichment Analysis of DEGs

**Figure 6:**
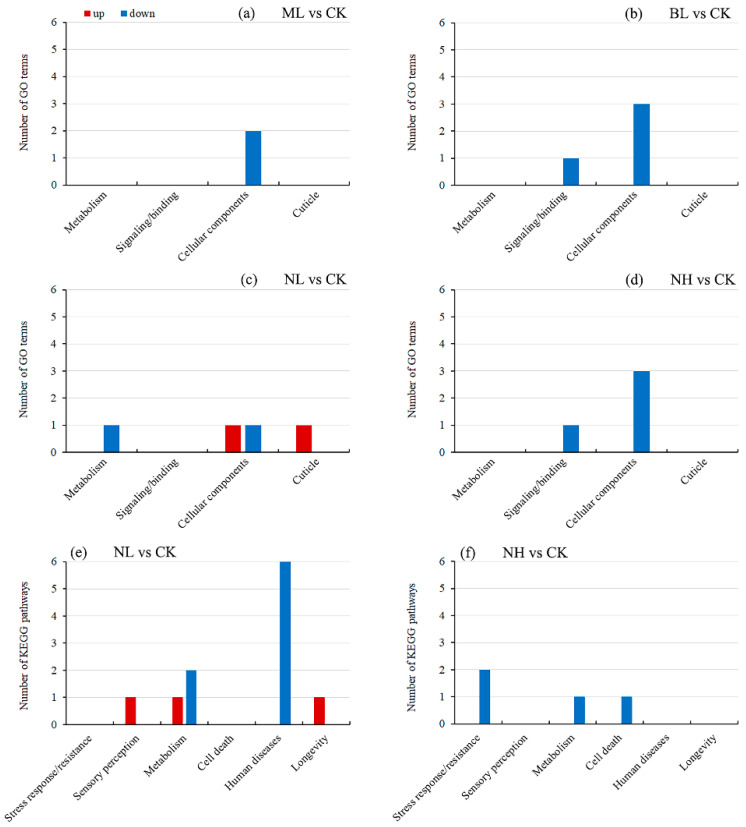
Enriched GO terms and KEGG pathways. (**a**–**d**) represent GO terms of ML vs. CK, BL vs. CK, NL vs. CK, and NH vs. CK groups, respectively. (**e**,**f**) represent KEGG pathways of NL vs. CK, and NH vs. CK groups, respectively (no pathways were significantly enriched in the ML vs. CK, and BL vs. CK groups). Red columns denote terms/pathways significantly enriched in the upregulated DEGs, while blue columns denote terms/pathways significantly enriched in the downregulated DEGs.

All DEGs were enriched to GO terms and KEGG pathways, resulting in 14 significantly enriched terms and 15 significantly enriched pathways (*q* < 0.05; Tables S7 and S8), which were functionally clustered into 9 distinct collections for a better understanding (Figure 6). Collectively, in the ML vs. CK, BL vs. CK and NH vs. CK groups, only downregulated DEGs were enriched to several terms, mainly associated with cellular components. In the NL vs. CK group, upregulated DEGs were enriched to one cuticle and one cellular component-related term, and downregulated DEGs were enriched in one metabolism and one cellular component-related terms. No pathways were significantly enriched in the ML vs. CK, BL vs. CK groups. In the NL vs. CK group, upregulated DEGs were enriched in one sensory perception, one metabolism and one longevity-related term, and downregulated DEGs were enriched in two metabolism and six human disease-related terms. Only a few terms were enriched by downregulated DEGs in the NH vs. CK group.

Notably, many cellular components related terms were enriched by a large number of DEGs, such as GO:0110165 (cellular anatomical entity) and GO:0016020 (membrane), which were enriched by 53–80 DEGs in both the BL vs. CK and NH vs. CK groups. Relatively more upregulated DEGs were enriched in a cuticle-related term, namely GO:0042302 (structural constituent of cuticle), which was enriched by 32 DEGs in the NL vs. CK group. For KEGG enrichment, relative fewer (3–5) DEGs were enriched in all pathways. In the NL vs. CK group, three upregulated DEGs were enriched to the longevity regulating pathway—worm (map04212). To further understand the response of this pathway to noise stress, all the genes related to this pathway were identified (127 genes; Table S9) and their expression pattern were illustrated by expression heatmap analysis (Figure S4). The Log2FC of these genes ranged from −2.4973 to 7.8006, while only three of them were identified as DEGs (LOC118267621, LOC118280550, and LOC118280298, fatty acyl-CoA reductase related genes; Table S9). Although not significant (*q* > 0.05), some key genes of the longevity pathway also showed some changes, such as downregulation of LOC118271659 (serine/threonine-protein kinase mTOR) and LOC118271657 (serine/threonine-protein kinase Tor), and upregulation of LOC118281031 (cholesterol 7-desaturase nvd).

#### 3.3.4. Clustering and Expression Heatmap Analysis of DEGs with Highly Significant Changes

DEGs with highly significant changes (*q* < 0.05, |log2FC| > 3) were further identified and analyzed. In the ML vs. CK, BL vs. CK, NL vs. CK, and NH vs. CK groups, 27 (1 up, 26 down), 120 (0 up, 120 down), 46 (29 up, 17 down), and 84 (13 up, 71 down) such DEGs were detected, respectively (Figure S5). Based on the above annotation and classification, the annotated ones of these DEGs were clustered into nine distinct collections (Figure S6). In the ML vs. CK group, very few (≤3) DEGs were assigned to each collection. In the BL vs. CK group, more DEGs (3–11) were assigned to each collection. In NL vs. CK group, a large number (17) of DEGs were assigned to cuticle, whereas very few (≤4) were assigned to other collections. In the NH vs. CK group, the highest number of DEGs (seven upregulated and four downregulated) were assigned to cuticle, and fewer (≤4) DEGs were assigned to other collections.

**Figure 7:**
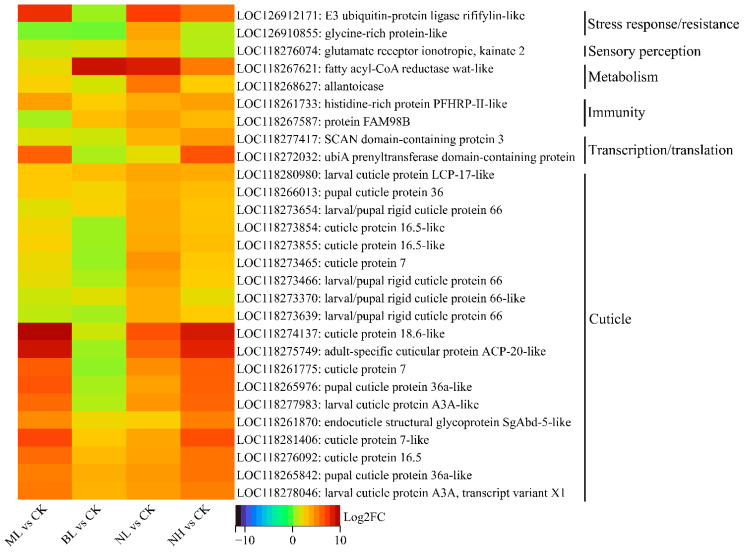
Expression heatmap of upregulated DEGs with *q* < 0.05 and log2FC > 3.

**Figure 8:**
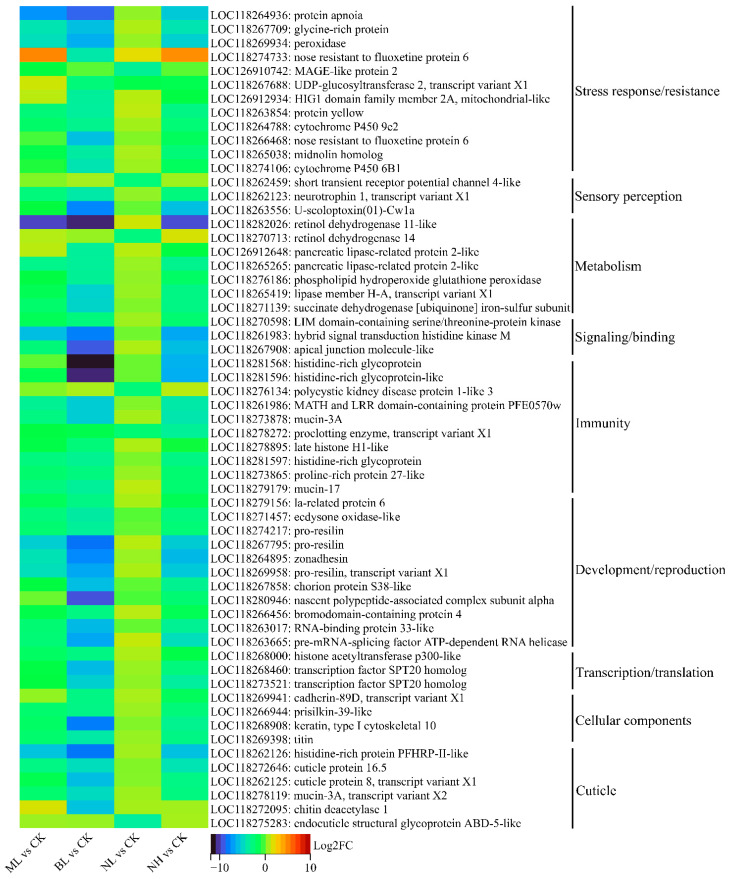
Expression heatmap of downregulated DEGs with *q* < 0.05 and log2FC < −3.

To check the expression pattern of these highly changed DEGs in all the four groups, their expression profiles were analyzed and illustrated by heatmaps (Figure 7 and Figure 8). It is showed that most of these upregulated DEGs were shown an upregulation trend in all groups (Figure 7). Such as many cuticle-related DEGs (e.g., LOC118281406: cuticle protein 7-like, and LOC118274137: cuticle protein 18.6-like), they are significantly (*q* < 0.05) upregulated in the NL vs. CK group; they also showed an obvious upregulation trend although not significant (*q* > 0.05) (Figure 7). Similarly, most of those identified downregulated DEGs maintained a downregulation trend in all the four groups (Figure 8).

### 3.4. RNA-Seq Validation

**Figure 9:**
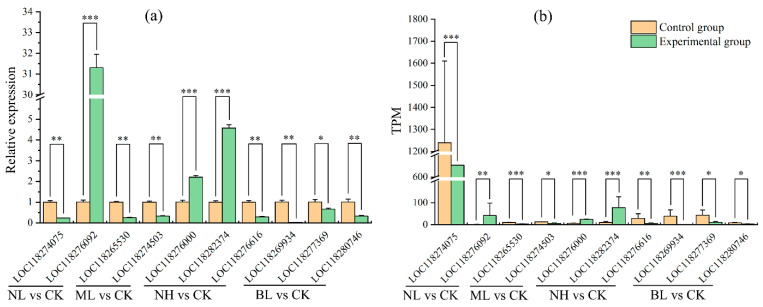
Validation of RNA-seq data by qPCR. (**a**) Relative expression levels of target genes determined by qPCR, * *p* < 0.05, ** *p* < 0.01, *** *p* < 0.001; (**b**) relative expression levels of target genes determined by RNA-seq, * *q* < 0.05, ** *q* < 0.01, *** *q* < 0.001.

To validate the accuracy of RNA-seq, 10 DEGs were selected from the four comparison groups for verification. The expression levels of the target genes determined by qPCR (Figure 9a) were consistent with those derived from RNA-seq analysis (Figure 9b), which confirmed the reliability of the RNA-seq results.

## 4. Discussion

In Lepidoptera, adult tympanal ears show anatomical and morphological diversity but share similar thresholds, optimally sensitive to 20–60 kHz ultrasound (bat echolocation range) [10,11], larvae and pupae lack tympanal organs but may perceive sound via non-specialized sensilla [12,13]. For instance, in *S. exigua*, bat echolocation signals significantly affected the activity and reproduction of adults while they altered larval metabolic processes [17]. And in *S. frugiperda*, exposure to prolonged bat calls resulted in a broad transcriptional response in adults [37]. In the present study, we further tested whether audible sound (250 Hz to 8 kHz) can affect the behavior of *S. frugiperda*. Results showed that all tested sounds exerted a suppressive effect on adult activity, with a dose-specific response trend following the order of 80 dB music < 120 dB music < 80 dB bird chirp < 80 dB noise < 120 dB bird chirp < 120 dB noise; in larvae, interestingly, low-intensity (80 dB) bird chirp and noise promoted their activity, whereas high-intensity (120 dB) bird chirp and noise suppressed their activity (Figure 1). This finding suggests that both larvae and adults of *S. frugiperda* are sensitive to these audible sounds.

We thus further tested whether and how persistent (for three successive generations) sound exposure (80 dB music, bird chirp and noise, and 120 dB noise; played either during the dark cycle or light cycle) can affect the fitness of *S. frugiperda* by measuring development and survival of larvae and pupae, as well as reproductive success of adults. Results showed that bird chirp (80 dB) and noise (80 and 120 dB) significantly negatively affected the fitness of *S. frugiperda*, and showed obvious dose-specific effects as 120 dB noise had greater effect than 80 dB noise and bird chirp, whereas 80 dB music usually showed no significant effect and even positive effect in some cases (Figure 2, Figure 3 and Figure 4). Similarly, previous studies in insects generally found that persistent acoustic (audible and ultrasonic) stressor exposure typically impairs their developmental fitness, survival rate and reproductive success [11,15,16,17]. In the case of the pink stalk borer, ultrasonic treatment exerted a marked inhibitory effect on multiple biological parameters of the pest’s immature stages, leading to substantial reductions in longevity, body weight, survival rate and fecundity [16]. While the intrinsic mechanisms driving insect responses to acoustic stress have not yet been fully clarified, sound stress exposure is known to reduce food intake and larval metabolism [17,23], disrupts mating decisions and mate choice [25], interferes with orientation and social interaction [24], as well as induces other biological and molecular changes [35,48], which thus may ultimately impair the survival and reproduction of insects. These results suggested that audible acoustic stressors also can be used as repellents to manipulate *S. frugiperda*. Moreover, our study showed that exposure of *S. frugiperda* to the sound stressors during the dark cycle or light cycle exerted similar effects (Figure 2, Figure 3 and Figure 4), which broadens its future use as a repellent. However, further validation is required before translating these findings into practical acoustic pest control strategies, including field exposure mapping, non-target risk assessment, and comprehensive dose–response modeling. In addition, acoustic conditions at insect positions in this study remain insufficiently characterized owing to equipment limitations; we have therefore provided acoustic waveform files (.wav) as supplementary data to support further research.

In the present study, we show that 80 dB bird chirp and noise prolonged the developmental duration of larvae and pupae, whereas 120 dB noise reduced these parameters, while all these sound stresses all reduced the bodyweight of larvae and pupae. Moreover, bird chirp and noise stresses promoted fecundity but reduced egg hatching, and resulted in fewer offspring gain. In *S. exigua*, similarly, different bat echolocation call stresses showed some fluctuate effects on the development and bodyweight of larvae and pupae, and adult lifespan and reproductive fitness [17]. It is still unknown how acoustic stimuli induced such consequence. In *S. frugiperda*, a previous study showed that the developmental periods of *S. frugiperda* larvae and pupae were significantly shortened after high-temperature selection [31]. Elevated temperatures enhance insect growth primarily by boosting metabolic rates, increasing enzyme activity (e.g., digestive and respiratory enzymes), and accelerating energy production [49]. Previous studies in insects and other animals also showed that biotic (such as environmental temperature and drought) and abiotic (such as pathogen) stresses can affect the egg number and quality, and there exist tradeoffs between egg size and egg number [50,51,52]. However, whether high intensity of sound stress follows such mechanisms needs further research.

It is noteworthy that research spanning multiple insect taxa has revealed that survival rates increase progressively under sub-lethal stress (such as temperature, drought and pesticide) after several generations of exposure, highlighting the occurrence of transgenerational acclimation [29,30,53,54]. For example, in the rice leaf roller *Cnaphalocrocis medinalis*, multigenerational heat selection enhanced larval heat tolerance [29]. In *S. frugiperda*, multigenerational exposure to high and low temperatures enhanced thermal and cold acclimation, respectively [31,55]. Acoustic adaptation has also been observed in *Gryllus bimaculatus* crickets: males near highway edges lower their chirp rate when passing cars approach, suggesting that repeated anthropogenic noise exposure may alter their noise sensitivity and behavioral responses, allowing them to maintain effective signaling rates [56]. In the present study, no obvious acoustic adaptation was observed in *S. frugiperda* following three successive generations of acoustic treatment applied across its entire life cycle, with consistent responses recorded across all trials (Figure 2, Figure 3 and Figure 4; no significant differences were observed among generations under the same treatment in most cases). However, additional studies are required to elucidate this issue in this moth species, including prolonged exposure across more generations with independent cohort replication [55] and detailed measurements of behavioral and physiological traits [17].

To test the underlying mechanisms of acoustic stress-induced effects, a few studies have tested gene transcription changes in locusts and other insects [35,36,37,48]. In the present study, RNA-seq analysis of larval whole body revealed that, compared to CK (silence), music induced fewer DEGs (71 in total, 11 up and 60 down), bird chirp induced much more DEGs (220 in total, 9 up and 211 down); noise also induced a great number of DEGs, particularly many more upregulated DEGs (100 and 64 for 80 dB and 120 dB noise, respectively). These results reflected that bird chirp and noise exerted greater stress on *S. frugiperda* larvae.

A prior study on *S. frugiperda* found that exposure to bat ultrasonic calls (vs. silence) altered male brain gene expression: 290 DEGs (with half annotated) were identified, including one heat shock protein and one antioxidant enzyme, with key enriched GO terms of chromatin binding, macromolecular complex binding and glutamate metabolic processes [37]. In our study, the majority (>80%) of DEGs were successfully annotated and were functionally clustered into 10 distinct collections (Figure 5). Among them, it is remarkable that all the 38 cuticle-related DEGs from NL vs. CK, and most (25/47 = 53.2%) such DEGs from NH vs. CK, were upregulated. In contrast, the majority of the remaining DEGs were downregulated, with most of them being involved in transcription and translation, immunity, development and reproduction, as well as stress response and resistance. Consistently, most (19/28) of the highly upregulated DEGs (*q* < 0.05, log2FC > 3) were associated with cuticle, while the highly downregulated DEGs (*q* < 0.05, log2FC < −3) were mainly associated with stress response/resistance (12/59), development/reproduction (11/59), immunity (10/59), and metabolism (7/59). Similarly, in locusts’ abdominal tympanic Müller’s organs, the majority of noise-induced transcripts with elevated abundance corresponded to genes responsible for cuticular biogenesis, suggesting extensive remodeling of partial or complete cuticular components within the auditory structure [36]. Increased expression of cuticular homologous in different termite castes was associated with greater thickness and hardness of the endocuticle [57]; thus, noise exposure probably induced the formation of a thicker and harder tympanum/cuticle in adults and larvae. In *S. frugiperda*, thermal pressure was found to trigger the upregulation of genes associated with cuticular development [31]. In *Aedes aegypti*, histological analysis of larval cuticular sections demonstrated that insecticide-selected strains exhibited a significantly thicker cuticle [58]. These lines of evidence suggested that a thicker, harder cuticle constitutes both a stress-responsive phenotype in insects and a trait analogous to stress resistance. However, linking whole-body DEG patterns to tissue-specific remodeling (e.g., tympanal or cuticular hardening) remains necessary [36]; further histological analyses, tissue-specific RNA profiling, or proteomic studies are also required to substantiate structural interpretations. Noise exposure also induces metabolic stress in *Drosophila* [59], and in locust auditory neurons, transcripts with reduced abundance are mostly involved in lipid and protein storage and metabolism [36], suggesting that excessive acoustic stimulation elicits a marked reduction in metabolic activity in insects.

In the present study, GO and KEGG enrichment analyses (Figure 6) further revealed remarkable differences between the different sound exposure groups. In the ML vs. CK, BL vs. CK, and NH vs. CK groups, only downregulated DEGs were enriched in several terms and pathways, which were mainly associated with cellular components. In the NL vs. CK group, upregulated DEGs were enriched in terms/pathways related to the cuticle, cellular components, sensory perception, metabolism, and longevity, while downregulated DEGs were mainly enriched in those associated with metabolism and human diseases. Furthermore, terms related to cellular components and the cuticle were enriched by substantially more DEGs (up to 80), further confirming the crucial role of the cuticle in sound stress resistance. In locusts, noise induced DEGs in auditory structures that were also associated with human disease-related pathways [36]. In *C. medinalis*, DEGs from larvae after heat stress were enriched to pathways related to longevity regulating, immunity, and diseases [60]. Similarly, KEGG enrichment analysis of heat stress-induced DEGs in *S*. *frugiperda* showed that many upregulated genes were associated with human diseases pathways and exhibited dose-specific effects, as their abundance increased with prolonged stress duration [31]. In the present study, DEGs in NL vs. CK group also enriched in a longevity regulating pathway (map04212: longevity regulating pathway—worm). Previous studies have suggested that lifespan extension via this pathway relies on four primary mechanisms: suppressed TOR signaling, DAF-16/FOXO regulation, enhanced steroid signaling via DAF-36/DAF-9/DAF-12, and elevated NHR-80/HNF-4 activity [61]. Although not statistically significant, several key genes of this pathway exhibited expression alterations in the NL vs. CK group. Specifically, LOC118271659 (serine/threonine-protein kinase mTOR) and LOC118271657 (serine/threonine-protein kinase Tor) were downregulated, whereas LOC118271657 (NVD, DAF36) was upregulated. Therefore, the sound stress-induced reduction in metabolism and development, as well as activated lifespan regulation, may serve as a key indicator of stress costs. Moreover, the mechanism underlying potential sound stress-induced lifespan regulation warrants further investigation.

In conclusion, this study demonstrated that high-intensity sound suppressed larval and adult activity, whereas low-intensity sound promoted larval movement. Multigenerational exposure revealed that bird chirp and noise, particularly high-intensity noise, significantly impaired developmental and reproductive fitness. Transcriptomic profiling demonstrated that sound stress suppressed genes linked to metabolism and immunity yet enhanced the expression of cuticle-associated genes. Taken together, audible sound elicited clear dose-dependent physiological and molecular responses in *S. frugiperda*. This study clarifies the multifaceted acoustic responses and related mechanisms of *S. frugiperda*, deepening our understanding of insect auditory sensitivity and providing a scientific basis for developing sound-based pest control strategies.

## Data Availability

The transcriptome raw reads have been deposited to the NCBI SRA database; the accession number is PRJNA1439234. The original contributions presented in this study are included in the article/Supplementary Material. Further inquiries can be directed to the corresponding authors.

## Supplementary Materials

The following supporting information can be downloaded at: https://www.mdpi.com/article/10.3390/insects17050467/s1, Figure S1. The principal component analysis (PCA) for RNA-seq of biological replicates of *S. frugiperda*. Figure S2. Volcanic plots of all DEGs. (a-d) represent DEGs of ML vs. CK, BL vs. CK, NL vs. CK, and NH vs. CK groups, respectively. Green dots indicate significantly downregulated DEGs; red dots indicate significantly upregulated DEGs; and blue dots indicate no significant differential expression. Figure S3. Venn diagram of DEGs. The overlapping regions of the circles denote the common DEGs across the combinations. Figure S4. Expression heatmap of genes related to the longevity regulating pathway—worm (map04212) within the NL vs. CK group. Figure S5. Volcanic plots of DEGs with highly significant changes (*q* < 0.05, |log2FC| > 3). (a-d) represent DEGs of ML vs. CK, BL vs. CK, NL vs. CK, and NH vs. CK groups, respectively. Green dots indicate significantly downregulated DEGs, and red dots indicate significantly upregulated DEGs. Figure S6. Annotation and classification of DEGs with highly significant changes (*q* < 0.05, |log2FC| > 3). (a–d) represent DEGs of ML vs. CK, BL vs. CK, NL vs. CK, and NH vs. CK groups, respectively. Red columns denote the significantly upregulated DEGs, while blue columns denote the significantly downregulated DEGs. Table S1. Sound pressure level (SPL) and frequency variability across cages (for adults) and boxes (for eggs, larvae and pupae) (the cage or box was placed 0.5 m directly in front of the speaker). Table S2. Primers for qPCR. Table S3. Effect of sound exposure on the behavior and fitness of *S. frugiperda*. Table S4. Summary of the quality of all sample sequencing data. Table S5 Pearson’s correlation coefficient. Table S6. Sound exposure induced DEGs. Table S7. GO enrichment analysis results. Table S8. KEGG enrichment analysis results. Table S9. Genes related to the longevity regulating pathway—worm (map04212). Audio File S1. Music (piano music, One Summer’s Day, Joe Hisaishi). Audio File S2. Bird chirp (recorded sparrow chirps). Audio File S3. Noise (recorded sound of a ceramic basin rubbing against the concrete floor).
