# Supplementary figures and images for: Audible Sound Stress Alters Behavior and Gene Transcription, and Negatively Impacts Development, Survival and Reproductive Fitness in Spodoptera frugiperda
Source: Insects. 2026 Apr 30;17(5):467. doi: 10.3390/insects17050467 (PMC13207539; doi:10.3390/insects17050467)

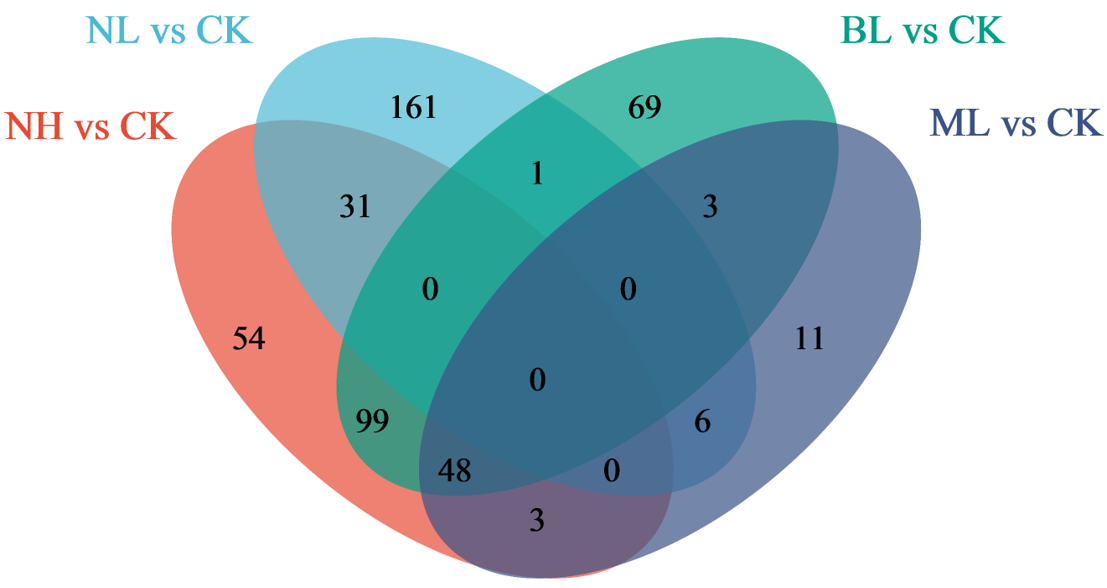

Supplement: Supplementary file 1 [file insects-17-00467-s001.zip › Fig. S3.tif]

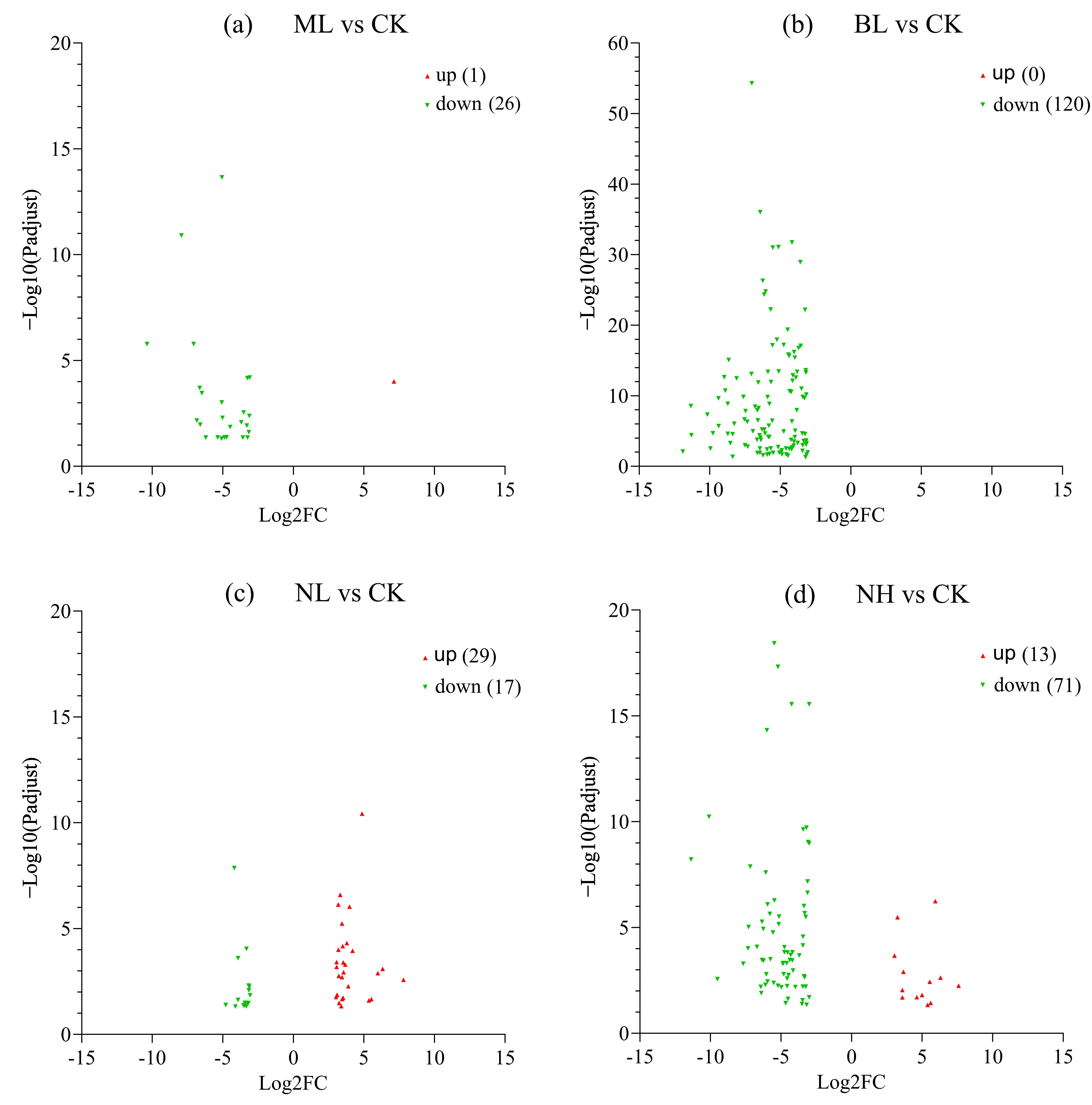

Supplement: Supplementary file 1 [file insects-17-00467-s001.zip › Fig. S5.tif]

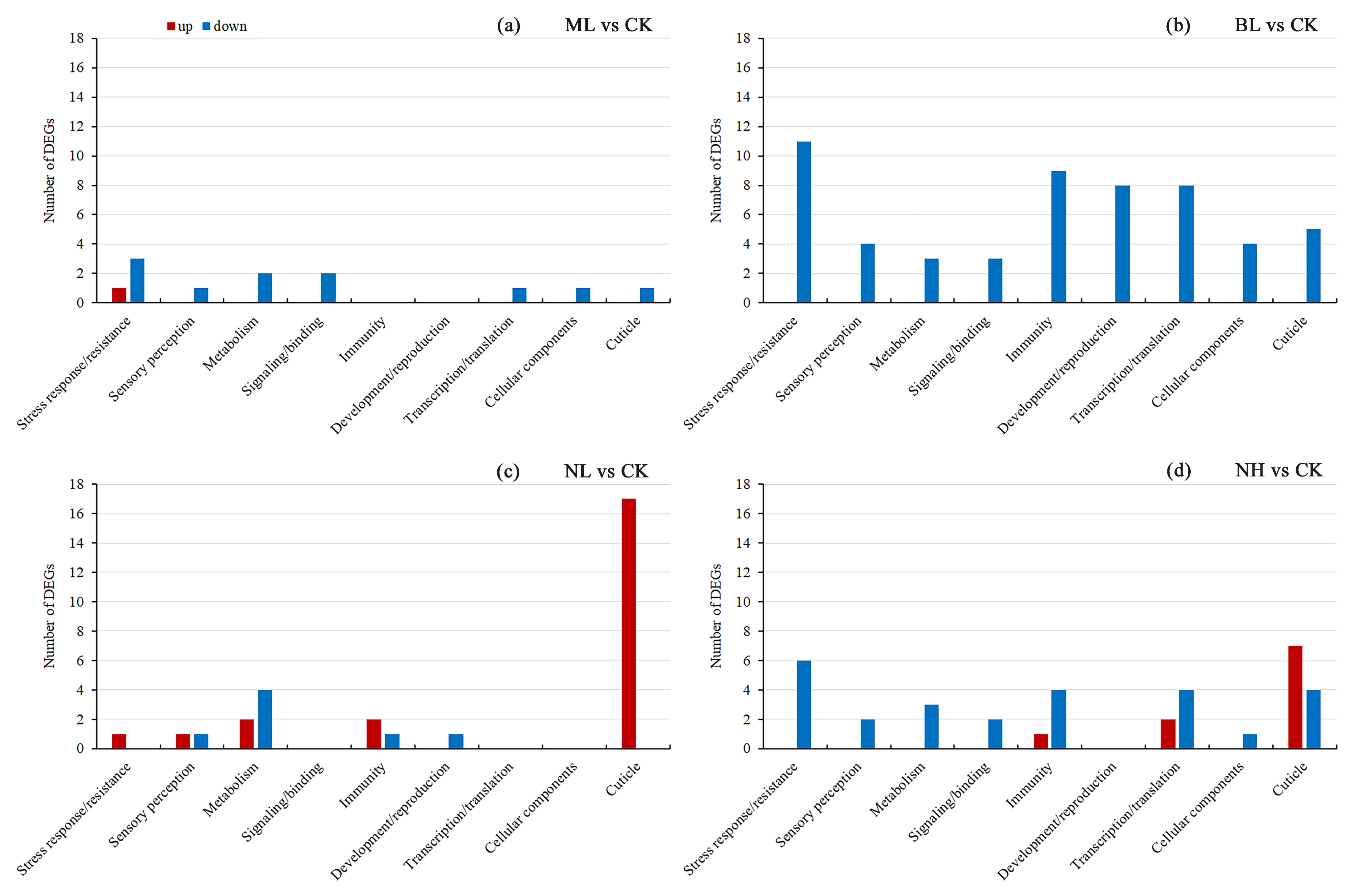

Supplement: Supplementary file 1 [file insects-17-00467-s001.zip › Fig. S6.tif]
